# Inhibition of Quorum Sensing Controlled Virulence Factors and Biofilm Formation of Streptococcus mutans Isolated From Orthodontic Subjects by 4-Hydroxycinnamic Acid

**DOI:** 10.7759/cureus.47490

**Published:** 2023-10-22

**Authors:** Kavitha Ramsundar, Ravindra Kumar Jain, Sankar G Pitchaipillai

**Affiliations:** 1 Orthodontics and Dentofacial Orthopedics, Saveetha Dental College, Saveetha Institute of Medical and Technical Sciences, Saveetha University, Chennai, IND; 2 Microbiology, Saveetha Dental College, Saveetha Institute of Medical and Technical Sciences, Saveetha University, Chennai, IND

**Keywords:** biofilm, white spot lesion, microorganism, 4-hca, quorum sensing

## Abstract

Introduction

Dental plaque biofilms are a collection of microorganisms that are adhered to the tooth enamel surface. Inhibition of plaque biofilms is required to prevent dental caries and periodontitis and currently, there are many chemical and herbal products in use for inhibition of biofilms but with limited success.

Materials and methods

Dental plaque collection was done from subjects undergoing orthodontic therapy followed by isolation of *Streptococcus mutans*. Isolated* S. mutans* were subjected to disk diffusion assay with 4-HCA (baseline 10mg/mL) for the zone of inhibition and broth micro-dilution to evaluate the minimum inhibitory concentration (MIC) and sub-MIC. Crystal violet staining was done for biofilm inhibition assay.

Results

Growth of *S. mutans* was inhibited by 4-HCA at concentrations as low as 0.31 mg/mL. 4-HCA (40μL) inhibited the bacterial growth and a clear zone (15 mm) was observed. 4-Hydroxycinnamic acids treated culture showed progressive reduction in the biofilm production at the concentration of 0.01 mg/mL. The 4-HCA concentration as low as 4 mg and 2 mg has remarkably inhibited biofilm formation of 49.3% and 34.3%, respectively.

Conclusion

The anti-quorum sensing and anti-biofilm activity of 4-Hydroxycinnamic acid against *S. mutans* isolated from subjects undergoing orthodontic treatment showed a remarkable result.

## Introduction

The persistence of oral bacterial infections is significantly influenced by biofilms, a collection of microorganisms that are permanently adhered to the surfaces of host cells (dental caries, periodontics, gingivitis, etc. [1]. Biofilms are collections of bacteria that are held to abiotic and biotic surfaces by a self-secreted extracellular polymeric material (EPS) [2]. One of the main oral infections, *Streptococcus mutans* (*S. mutans*), can essentially alter the process of tooth enamel demineralization because of its acid production and tooth adhesion capabilities [3] S. mutans releases a variety of virulence agents, and the quorum sensing (QS) system regulates the production of biofilms [4]. The bacterial attachment to the tooth surface may be attributed to the production of virulence factors by them (Glucosyltransferases, acidogenesis, acid tolerance, and exopolysaccharide) thereby evading the host's innate immune system [5].

In bacteria, quorum sensing, also known as cell density-mediated gene expression, regulates the expression of certain genes by accumulating signaling chemicals that enable intercellular communication [6]. Quorum-sensing signaling is a type of signaling that is triggered in response to cell density [7]. The concentration of autoinducers is a function of microbial density since they are produced at a constant rate. The signal is perceived when its concentration reaches a certain level. Because a particular number of microorganisms must be present for the signal to be sensed and the population to respond to the signal, the term “quorum” is used to characterize this type of signal system [8].

Hydroxycinnamic acids (HCA) are polyphenols that include caffeic acid, isoferulic acid, coumaric acid, and ferulic acid present in fruits, vegetables, and coffee. They have antioxidant, antimicrobial, anti-inflammatory, and cytoprotective properties, which are used in the treatment of diseases such as cancer, chronic inflammation, neurodegenerative and cardiovascular disorders. There are two types: Natural or synthetic 4-Hydroxycinnamic acid (4-HCA). Natural 4-HCA is excreted as metabolites by some microorganisms like Phomopsis liquidambari or extracted from reproductive flowers, seeds and leaves [9]. Hence this study was done to evaluate the anti-quorum sensing and anti-biofilm activity of 4-HCA against S. mutans isolated from subjects undergoing orthodontic treatment.

## Materials and methods

Study design and clinical isolates

Dental plaque samples were collected from the upper molar and lower incisor teeth of 5 patients undergoing orthodontic treatment with 0.022-inch MBT (McLaughlin, Bennett, Trevisi) metal brackets at the Department of Orthodontics, Saveetha Dental College, and hospitals. Plaque samples were collected using a sterile No. 23 Shepherd's hook explorer. The plaque samples were collected for isolation of the caries causing *S. mutans* in orthodontic patients. The collected samples were inoculated into Brain Heart Infusion (BHI) (HiMedia, India) broth and incubated for 24 hours at 37°C. The BHI broth samples were then inoculated in the Mutans-sanguis Agar (HiMedia) plates, which were then incubated for 24 hours at 37°C. For examination, based on the colony morphology *S. mutans* colonies were isolated and were stored at 4°C. Further, the *S. mutans* colonies were identified by a standard identification test [10].

Collection of synthetic compound

4-HCA was purchased from Sigma-Aldrich (St. Louis, MO). 

Disk diffusion assay

In accordance with the Clinical and Laboratory Standards Institute (CLSI) Guidelines, the Kirby Baur Disk diffusion method was used to conduct the antibiotic susceptibility test [11]. The lawn culture of *S. mutans* was done on a Mueller-Hinton agar (HiMedia) plate. Wells were cut in the plate using a well-cutter. 40μL of 4-HCA and sterile water (control) was added into the separate wells and incubated at 37°C for 24 hours. A zone of inhibition was recorded. 

Determination of minimum inhibitory concentration (MIC)

According to previously published literature, the MIC of 4-HCA were determined [12] and Methanol (Sigma-Aldrich) was used as the solvent for 4-HCA. Briefly, 10μL of the *S. mutans* broth culture with the cell mass equivalent to 0.5 McFarland turbidity standard units (1.5 × 10^8^ CFU/mL) was added to tubes containing Luria Bertani (LB) (HiMedia) broth and serially diluted (two-fold) to attain final concentrations starting from 0.01875 to 9.6 mg/mL. Similarly, 4-HCA was added (10-0.015 mg/mL) to LB broth tubes containing *S. mutans*. All the tubes were incubated (shaker incubator) at 37 °C for 24 h. The growth of the 4-HCA-treated *S. mutans* was compared with that of an untreated control by measuring the bacterial cell density at 600 nm (data not shown). For further confirmation, 10μL of 2,3,5-triphenyl tetrazolium chloride (TTC) was added to the tubes, which were observed for a cherry-red color change. The lowest concentration with no visible growth of *S. mutans* was recorded as MIC. Further, the anti-biofilm experiments were performed at the sub-MIC concentrations of 4-HCA.

Biofilm inhibition assay

Using a standard micro-titer plate test, the impact of the 4-HCA on *S. mutan's *ability to produce biofilms was investigated. Briefly, tryptic soy broth (TSB) (190 mL) with or without 4-HCA (starting from 0.15mg/mL) was dispensed into the wells of a 96-well microtiter plate with an overnight culture of the test *S. mutans* strain (OD adjusted to 0.4 at 600 nm using spectrophotometry (Nunc, Japan). Without agitation, the plate was incubated at 37 °C for 24 hours before being cleaned three times with deionized water. After dissolving crystal violet in 95% ethanol, the absorbance was measured at 595 nm as previously mentioned [13,14].

## Results

MICs of 4-HCA on *S. mutans*

The broth microdilution method was used to assess the MIC of 4-HCA against *S. mutans*. Growth of *S. mutans* was inhibited by 4-HCA at concentrations as low as 0.31 mg/mL (Table 1).

**Table 1:** MIC assay for 4-Hydroxycinnamic acid against Streptococcus mutans MIC - Minimum Inhibitory Concentration

S.no.	Synthetic compound	Organism	Conc./mL	MIC
1	4-Hydroxycinnamic acid	Streptococcus mutans	10 mg	MIC
2	4-Hydroxycinnamic acid	Streptococcus mutans	5 mg	MIC
3	4-Hydroxycinnamic acid	Streptococcus mutans	2.5 mg	MIC
4	4-Hydroxycinnamic acid	Streptococcus mutans	1.25 mg	MIC
5	4-Hydroxycinnamic acid	Streptococcus mutans	0.62 mg	MIC
6	4-Hydroxycinnamic acid	Streptococcus mutans	0.31mg	MIC
7	4-Hydroxycinnamic acid	Streptococcus mutans	0.15 mg	Growth
8	4-Hydroxycinnamic acid	Streptococcus mutans	0.07 mg	Growth
9	4-Hydroxycinnamic acid	Streptococcus mutans	0.03 mg	Growth
10	4-Hydroxycinnamic acid	Streptococcus mutans	0.015 mg	Growth

For additional research, 4-HCA concentrations below the MIC were employed to test for anti-biofilm activity against *S. mutans*.

Zone of inhibition

**Figure 1:**
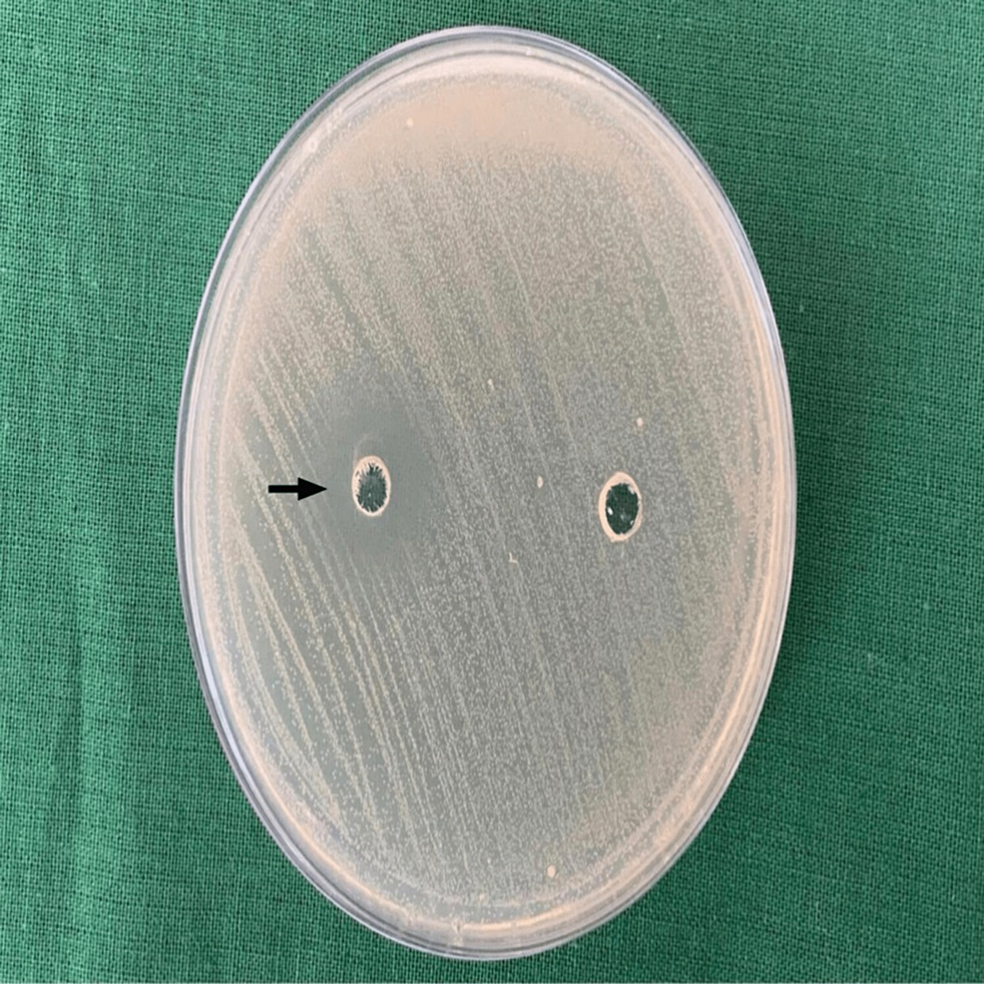
Three-dimensional disk diffusion test shows the zone of inhibition for 4-HCA.

Disk diffusion test of 4-HCA (40μL) and negative control (sterile water) against *S. mutans* shows a zone clear zone of inhibition of 15mm indicating anti-bacterial activity against *S. mutans* (Figure 1).

Crystal violet staining

**Figure 2:**
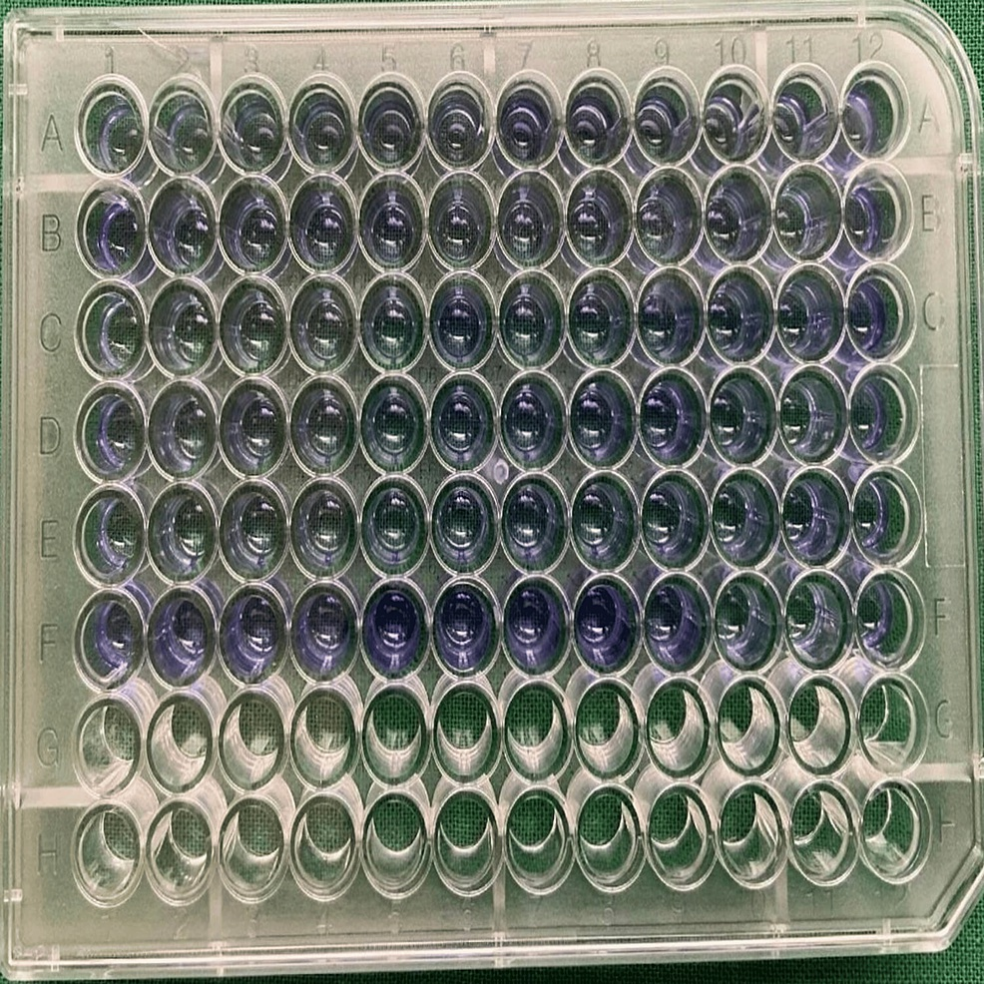
Biofilm formation assessed with crystal violet staining.

4-HCAs treated culture showed progressive reduction in the biofilm production till the concentration of 0.01 mg/mL (Figure 2).

Effect of synthetic compound (4-HCA) on biofilm formation in *S. mutans*

**Figure 3:**
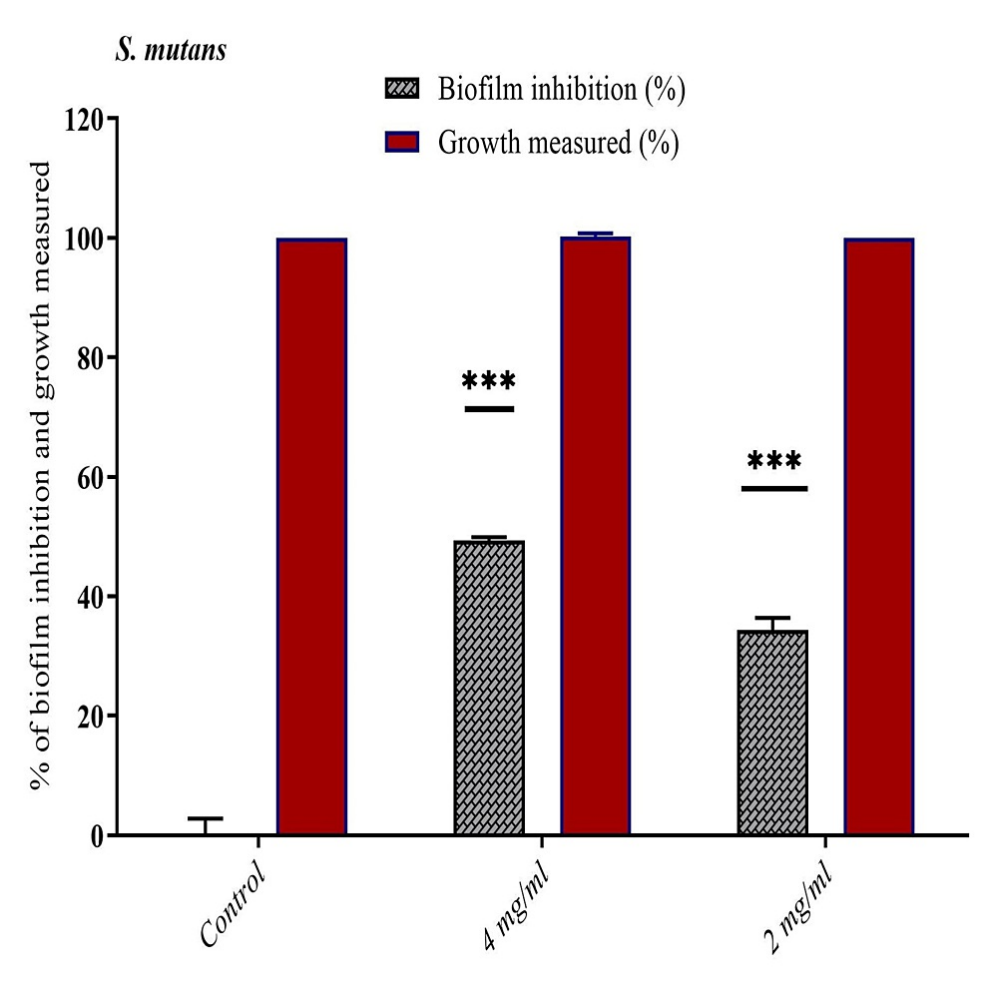
Bar chart showing the anti-biofilm activity of 4-Hydroxycinnamic acid against S. mutans under different concentrations.

Noticeable reduction in biofilm formation was observed when the organisms were grown in the presence of 4-HCA. The 4-HCA concentration as low as 4 mg and 2 mg has remarkably inhibited biofilm formation of 49.3% and 34.3%, respectively (Figure 3).

## Discussion

*S. mutans* is most commonly implicated in the pathogenesis of dental caries and enamel white spot lesions in subjects undergoing orthodontic treatment. These subjects harbor *S. mutan*s in dental plaque as oral hygiene maintenance is challenging due to the presence of appliances thereby making the teeth susceptible to WSLs. Prevention and management of WSLs is an integral part of orthodontic treatment and many agents have been used in the past with limited success. In the present study, an investigation of 4-HCA for its in vitro effects of Quorum sensing-controlled virulence factors and biofilm inhibitory activities against *S. mutans* was done. It is crucial to find compounds that inhibit QS, which limit bacterial growth in order to avoid medication resistance. Agents that reduce bacterial virulence without encouraging resistance could potentially offer significant therapeutic benefits. It is possible that virulence attenuation will have significant therapeutic benefits without developing resistance [15]. In the present study, we determined the MIC of 4-HCA against *S. mutans* with the broth microdilution method. Growth of *S. mutans* was inhibited by 4-HCA at concentrations as low as 0.31 mg/mL and higher concentrations resulted in no inhibition. A zone of inhibition of 15 mm was established for 40μL of 4-HCA in this study. At concentrations as low as 4 mg and 2 mg/dL 4-HCA has remarkably inhibited biofilm formation. With these significant findings, we can conclude that 4-HCA has a remarkable anti-bacterial and anti-biofilm activity against *S. mutans* and hence can be therapeutically used for the prevention and treatment of dental caries and white spot lesions.

Cinnamic acid and its structural derivatives are phenolic acids that are lipophilic and have potent antioxidant effects they act as chelators and scavengers of free radicals demonstrating inherent antimicrobial activity [16]. Cinnamic acid and its derivatives have proven anti-microbial against *M. tuberculosis* and are proven to inhibit the fatty acid synthase type II which is essential for the organism’s survival [17]. Previous literature on the effect of cinnamic acid derivatives against certain bacteria is available but since there are no published papers on the effect of 4-HCA on *S. mutans* the present study was done. According to the study by Lavertyet al*., *cinnamic peptides had an increased activity against* S. aureus* and methicillin-resistant *Staphylococcus aureus* [18]. A study by Zhou et al. reported on the anti-quorum sensing and anti-biofilm activity of 4-HCA against *Agrobacterium tumefaciens* [19]. The anti-QS potential of HA was evaluated by β-galactosidase assay and acylated homoserine lactones (AHL) analysis in their study whereas in the present study crystal violet staining was used. They found that the MIC of 4-HCA against Agrobacterium tumefaciens was greater than 1.20 mM. The development of biofilms dramatically decreased by 40%, 61%, and 83%, respectively, following treatment with 4-HCA at concentrations of 0.30 mM, 0.60 mM, and 0.80 mM [20]. Whereas in the present study, the growth of *S. mutans* was inhibited by 4-HCA at concentrations as low as 0.31 mg/mL.

Cheng et al.* *reported the effects of 4-methoxy cinnamic acid and dimethylamino cinnamic acid on biofilms as determined by crystal violet staining. The biofilms were significantly diminished by 42%, 48%, and 64% after exposure to 4-methoxy cinnamic acid at concentrations of 50, 100, and 200 g/ mL, respectively. Dimethylamino cinnamic acid was more effective than 4-methoxycinnamic acid and at concentrations of 25, 50, and 100 g/mL, respectively, the biofilms were reduced by 60%, 72%, and 75% [20]. But in the current study, 4-HCA inhibited the growth of *S. mutans* at MICs as low as 0.31 mg/mL and the concentration of 4 mg and 2 mg of 4-HCA had remarkably inhibited biofilm formation of 49.3% and 34.3% against *S. mutans*,* *respectively. This demonstrates its QS-inhibition and anti-biofilm properties.

In the present in vitro study, limited experimental methods were used to study the QS activity of 4-HCA. Being an in-vitro study in itself is a limitation and hence the synthetic compound should be subjected to further evaluation involving experimental animals and then human clinical trials must be conducted. The future scope includes further evaluation of the compound and exploring its potential for using it as an active compound for oral hygiene maintenance in subjects undergoing orthodontic treatment.

## Conclusions

The anti-quorum sensing and anti-biofilm activity of 4-HCA against *S. mutans* isolated from subjects undergoing orthodontic treatment showed a remarkable result and it inhibited growth of *S. mutans* at concentrations as low as 0.31 mg/mL. Hence, 4-HCA can be used as a potent anti-microbial agent against *S. mutans, *and it can be incorporated into oral prophylactic devices after further testing.
